# The impact of digital economy development on subjective well-being: micro-level evidence from China

**DOI:** 10.3389/fpubh.2025.1660857

**Published:** 2025-10-15

**Authors:** Hao Li, Jiao Li, Zihan Yang

**Affiliations:** ^1^School of Business, Xinyang Normal University, Xinyang, China; ^2^Research Institute of the Economic and Social Development in the Dabie Mountains, Xinyang, China

**Keywords:** digital economy, subjective well-being, digital divide, smart living environment, sense of happiness

## Abstract

**Introduction:**

In the era of digital transformation, exploring the welfare implications of societal changes and in particular the relationship between digital economy development and subjective well-being (SWB) has emerged as a critical focus of academic research. Clarifying how digital economy development affects SWB and its underlying mechanisms is essential for improving societal welfare and quality of life in the context of digitalization.

**Methods:**

This study employs empirical analysis to address the research gap. It combines two types of data: 1) micro-level data from the China Family Panel Studies (CFPS) covering the years 2018, 2020, and 2022; and 2) macro-level metrics of regional digital economy development. The integrated dataset is used to examine the impact of digital economy development on SWB and identify its intrinsic mechanisms.

**Results:**

Digital economy development significantly enhances residents’ SWB, with key transmission channels including the popularization of digital applications, improved information acquisition capabilities, and optimized digital living environments; meanwhile, heterogeneous regression analyses reveal that the positive impact of digital economy development on SWB varies significantly by age and frequency of digital technology usage, and mediation effect tests further confirm that the digital economy influences SWB primarily through five pathways, namely economic improvement, health enhancement, life quality upgrading, environmental optimization, and governance efficiency improvement.

**Discussion:**

Strengthening the construction of digital infrastructure, establishing tiered intervention mechanisms to address heterogeneous impacts across age and usage frequency groups, developing dynamic evaluation systems for digital economy welfare effects, and building a new digital governance paradigm that balances efficiency and fairness are targeted recommendations. These measures aim to fully leverage the welfare-enhancing role of the digital economy, ultimately promoting overall societal welfare and improving residents’ quality of life.

## 1 Introduction

From a national perspective, the core goal of economic progress and government policies is to enhance residents’ subjective well-being (SWB) and quality of life, thereby advancing societal welfare. Thus, SWB serves as a direct barometer of a nation’s overall welfare level. As social economies evolve, happiness remains the ultimate pursuit of human endeavors, and individual welfare extends beyond material prosperity to encompass subjective contentment. SWB, a pivotal dimension of personal welfare, refers to individuals’ holistic subjective evaluations of their current life circumstances—encompassing both cognitive judgments of living conditions (e.g., life satisfaction) and affective experiences (positive/negative emotions) (1). It has become a crucial aspect of personal welfare, representing individuals’ subjective assessments and emotional experiences related to their current circumstances (2). This range of feelings (from positive to negative) reflects individuals’ psychological well-being (PWB)—a broader construct that includes SWB, autonomy, and purpose in life (3). SWB, as the affective-cognitive core of PWB, directly mirrors individuals’ psychological adjustment to their life contexts (2).

The Chinese government has centered its mission in the new era on pursuing people’s well-being, with happiness as a key focus—particularly in the information technology field, where ensuring residents’ sense of progress, happiness, and security is paramount. As material needs are increasingly met, happiness has become a primary concern for the public. However, research highlights a “Happiness Paradox” in China: overall happiness has not kept pace with economic growth (4). According to the China Household Finance Survey (The China Family Finance Survey and Research Center), the proportion of happy families rose from 56.7% in 2013 to 70.2% in 2017, yet China’s happiness levels still lag behind many leading nations globally. The World Happiness Report (a collaboration between the United Nations and Columbia University’s Earth Institute) ranked China 72nd among 146 countries/regions in 2022—an improvement from 93rd in 2013, but still in the mid-range, leaving substantial room for enhancement (5, 6).

The implementation of national initiatives such as “Broadband China” and “Digital China” has accelerated the upgrade of China’s information infrastructure, fostering the rise of a vibrant digital economy driven by the internet, big data, artificial intelligence, and cloud computing. The scale of China’s digital economy has expanded from 22.6 trillion yuan in 2016 to 53.9 trillion yuan in 2023, and it has emerged as a core engine of national economic growth (7). Digital technology services have integrated into all aspects of production and daily life, strongly supporting the development of productive and living service sectors, and effectively meeting societal demand for improved quality of life (8).

However, three critical gaps remain: First, few studies establish causal links between the holistic digital economy (rather than individual digital tools) and residents’ SWB, limiting understanding of its systemic welfare effects (9, 10). Second, most literature relies on single-period cross-sectional or mixed cross-sectional data, which fail to address individual fixed effects or capture dynamic changes in SWB over time—leading to potential estimation bias (11). Third, existing analyses often adopt single-path explanations (e.g., economic or environmental channels) and lack exploration of heterogeneous effects or multi-dimensional mechanisms, restricting insights into how the digital economy shapes welfare for different groups (12).

To fill these gaps, this study integrates three waves of panel data (2018, 2020, 2022) from the China Family Panel Study (CFPS) with corresponding digital economy indicators. By constructing a dynamic panel model, we address endogeneity issues arising from individual heterogeneity, systematically examine how the digital economy influences SWB, and clarify its underlying mechanisms. This approach enhances the precision of causal inferences and provides empirical evidence for optimizing digital governance and improving societal welfare.

Relative to existing studies, this research offers four key marginal contributions: First, methodologically, building on the aforementioned data integration, it further controls for regional development differences (beyond individual heterogeneity) to mitigate endogeneity more comprehensively, and constructs a panel model to identify the effect of digital economy growth on residents’ SWB—providing a replicable template for digital welfare research in developing countries. Second, in variable measurement, it develops a multi-dimensional index for individual digital economy utilization (covering digital device usage, applications, dependency, information acquisition, and living environment), moving beyond single-indicator approaches to capture structural differences in digital technology’s welfare effects. Third, regarding mechanisms, it validates five transmission pathways (economic, health, life quality, environmental, governance) through which the digital economy boosts well-being, breaking the limitation of single-path explanations in existing literature. Fourth, in heterogeneity analysis, it explores subgroup differences, revealing group-specific patterns of digital welfare effects—laying a foundation for understanding digital-era welfare distribution and optimizing policies to reduce welfare inequality.

## 2 Theoretical framework and research hypotheses

To systematically analyze the influence of the digital economy on residents’ SWB, defined as individuals’ holistic perception and evaluation of their living conditions, including cognitive life satisfaction and emotional states (1), this study ​draws on​ two core theoretical frameworks that serve as a rigorous lens. First, the Capability Approach (13) posits that well-being originates from individuals’ ability to achieve “valued functioning” (e.g., stable income, good health, quality public services). For the digital economy context, this framework explains how digital technologies enhance such capabilities: by reducing information asymmetry (e.g., enabling remote access to job opportunities) and lowering transaction costs (e.g., facilitating telemedicine for rural residents), the digital economy expands the range of functioning that individuals can realize, thereby potentially boosting SWB. Complementing this, the Digital Divide Theory highlights disparities in digital access, skills, and usage as key barriers to equitable welfare distribution—this directly addresses why groups such as the older adults or low-educated individuals may experience heterogeneous SWB effects from the digital economy, laying the groundwork for subsequent heterogeneity analysis (14). Together, these two frameworks, paired with existing literature confirming SWB’s sensitivity to economic, environmental, and governance factors, form the basis for exploring how digital technology reshapes lifestyles and work patterns to influence happiness (15–17).

Existing research on the digital economy-SWB relationship, while accumulating insights, has left critical academic debates unresolved and details underexplored. First, the direct link between the two remains contested: the “Happiness Facilitation Theory” argues digital tools enhance SWB by improving daily convenience (e.g., e-commerce, online education), strengthening social connections via online communities, and boosting productivity in work and study (9, 18, 19). In contrast, the “Happiness Inhibition Theory” warns of negative effects such as information cocoons, upward social comparisons triggered by social media, and reduced offline interpersonal interaction (20–22). A key limitation of these debates is their overreliance on single digital tools (e.g., internet or mobile phone use) rather than the holistic digital economy, which fails to capture systemic welfare effects of digital transformation (9, 10). Second, while mediating pathways like economic (job creation, income equality), health (digital literacy, healthcare access), lifestyle (convenience, diverse services), and environmental (smart governance, green lifestyles) mechanisms have been identified, governance-related pathways—especially how digital government efficiency and public trust in digital governance moderate the digital economy-SWB relationship—remain largely unexamined (9, 11, 12, 15, 23). Thirdly, despite acknowledging the heterogeneous effects based on age (where middle-aged individuals tend to benefit more than minors or the older adults) and education (with those possessing higher education making better use of digital resources), the frequency and duration of digital technology usage have seldom been the focal points of targeted analysis, thereby constraining the breadth of existing conclusions (24).

### 2.1 The digital economy and residents’ income: the economic pathway

The digital economy integrates technologies like AI, cloud computing, and smart healthcare to revolutionize work and life, creating new jobs, improving employment rates, and elevating wages (15). It strengthens industrial chains, supports upstream and downstream sectors, and introduces new business models (e-commerce, e-learning, Internet+), while digital platforms enhance job market efficiency by simplifying job searches, expanding opportunity access, and raising incomes (25). Additionally, it reduces entrepreneurship costs and breaks time–space barriers, stimulating entrepreneurial activity, invigorating societal entrepreneurship, and increasing individual earnings and satisfaction (26). Digital technology also promotes information sharing, narrows income inequality via inclusive digital finance, cuts living costs, and bridges group gaps (11). Based on this, Research Hypothesis 1 (H1) is proposed: The growth of the digital economy improves residents’ SWB by increasing their incomes and reducing income inequality.

### 2.2 The digital economy and individual health: the health pathway

A healthy body is foundational to a fulfilling life, and the digital economy advances “digital health literacy”—skills to access, use, and create digital health resources—improving health information access, awareness of healthy activities, health knowledge, and healthy behaviors (23). Digital technology guides individuals to relevant health information, enhances the effectiveness of health messages, and improves fitness outcomes (27). It also transforms health consultations and services: digitizing health assessments, optimizing consultation efficiency, and health information management, and medical services, narrowing urban–rural healthcare gaps, and improving disease prevention (12). Furthermore, digital technology enhances government health decision-making and governance, providing more efficient health services and expanding health information accessibility (28). Thus, Research Hypothesis 2 (H2) is proposed: The digital economy improves residents’ SWB by enhancing their digital health literacy and healthcare accessibility.

### 2.3 The digital economy and quality of life: the quality-of-life pathway

The digital economy saves time and energy while boosting convenience and comfort, offering efficient, diverse services (online shopping, healthcare, education) to elevate quality of life and well-being (9). Online libraries and educational platforms provide extensive knowledge resources, enabling easy information access and horizon expansion (29). Social media and online communities facilitate interaction and information sharing, promoting socialization and equitable social engagement (18). Prevalent digital technologies have transformed consumption habits, enabling online leisure activities that increase leisure time and consumption, while digital tools enhance productivity and innovation to further support well-being (19, 30). Platforms for inclusive finance, telemedicine, education, and entertainment bridge information gaps, reduce transaction costs, and optimize asset allocation to improve daily life (31). Therefore, Research Hypothesis 3 (H3) is proposed: The digital economy improves residents’ SWB by enhancing their quality of life.

### 2.4 The digital economy and environmental quality: the environmental pathway

The environment is critical to survival—lush mountains and clear skies embody natural beauty and contentment—while pollution increases disease risk, endangers physical health, triggers anxiety/despair, harms mental well-being, and reduces overall happiness (32). The digital economy, with its openness and interactivity, offers real-time data access to revolutionize environmental decision-making, pioneer pollution management, and drive green growth (33). It enables regulators to access key environmental information, overcome traditional regulatory limitations, strengthen oversight, and encourage public participation in shaping environmental norms (16). Additionally, it promotes eco-friendly consumption (sharing economy, paperless operations, public transport, online learning) to minimize resource waste, optimize resource allocation, foster sustainable lifestyles, revitalize industrial structures, and enhance environmental responsibility (34). Hence, Research Hypothesis 4 (H4) is proposed: The digital economy improves residents’ SWB by optimizing environmental quality through smart governance and green lifestyles.

### 2.5 The digital economy and government effectiveness: the governance pathway

In China’s government-led economy, advancements in government quality (performance, political trust, efficiency, anti-corruption) significantly improve resident well-being (17). Translating well-being into tangible outcomes requires transforming government roles, innovating regulation, enhancing credibility, and ensuring policy benefits reach the public. Unlike traditional governance, digital government uses cutting-edge technology to improve public policy/service quality, expand communication, manage public opinion, streamline administrative processes, and create interactive discourse platforms—boosting effectiveness, efficiency, financial transparency, and social credibility (35). Digital technology’s openness, interactivity, and real-time responsiveness help the government understand citizen/business demands, while digital inclusivity amplifies diverse voices, strengthens taxpayer-public interaction, bridges the digital divide, and promotes equity (36). Strengthened digital governance and improved services better meet public needs, fostering social harmony and well-being. Based on this, Research Hypothesis 5 (H5) is proposed: The digital economy improves residents’ SWB by enhancing government effectiveness and public trust in government.

### 2.6 Heterogeneous effects of the digital economy across individual characteristics

**Figure 1:**
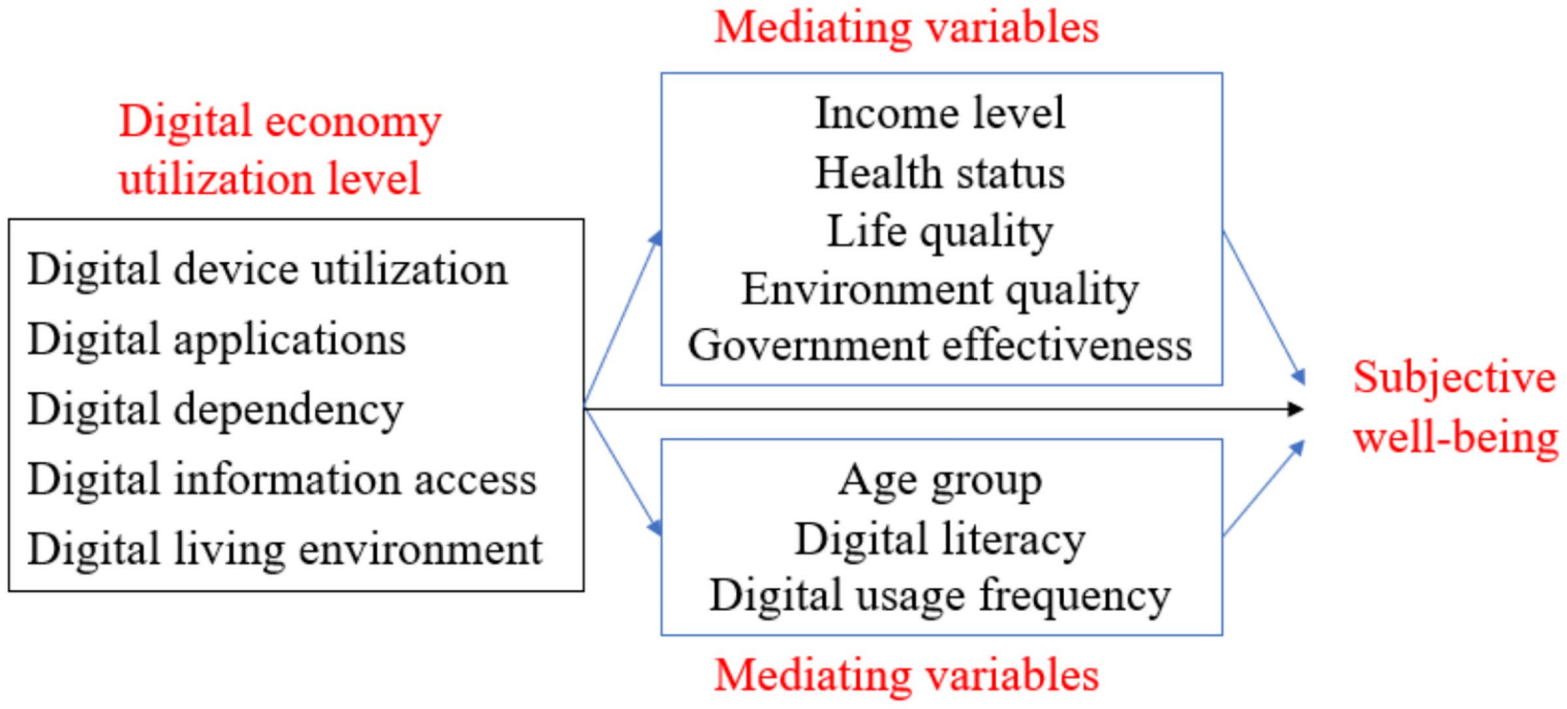
Conceptual framework of digital-economy effects on SWB. The framework clarifies the multi-dimensional transmission mechanism of the digital economy on SWB. The “individual level of digital economy utilization” includes five core dimensions (digital device utilization, digital applications, digital dependency, digital information access, digital living environment); “moderating variables” cover age group, digital literacy, and digital usage frequency, which regulate the strength of the digital economy’s impact on SWB; “mediating variables” (economic level, health status, life quality, environmental quality, government effectiveness) are the key channels through which the digital economy affects SWB.

While the digital economy offers broad benefits, it poses challenges for low-digital-literacy groups. In employment/income, digital technologies improve efficiency but increase job insecurity for low-IT-skill workers, and online consumption platforms squeeze traditional industries, raising unemployment/income decline risks for traditional workers (9, 25). In health, the “digital divide” and “information cocoon” harm well-being: excessive digital information fuels upward comparisons, underestimates actual status, and creates harmful psychological gaps; over-reliance on the internet reduces offline emotional interaction and social participation motivation; and prolonged digital entertainment use causes late nights, sedentarism, and poor health (12, 21, 22). Notably, the older adults face digital barriers (e.g., inability to use mobile payments), increasing daily difficulties (22). These differences indicate SWB effects vary by individual traits. Given the absence of direct digital literacy indicators in the CFPS dataset, we operationalize “digital literacy” using “education level” as a proxy, following Li and Yang (12); specifically, individuals with a college degree or above are categorized as “high digital literacy,” while those with middle school education or below are categorized as “low digital literacy.” Thus, Research Hypothesis 6 (H6) is proposed, with three sub-hypotheses (see Figure 1):

H6a: The positive effect of the digital economy on SWB is stronger for middle-aged adults (30–59 years) than for minors (<30 years) and the older adults (>60 years);

H6b: The positive effect is stronger for high-educated individuals (college degree or above) than for low-educated individuals;

H6c: The positive effect is stronger for high-frequency digital users than for low-frequency users.

## 3 Model specification and variable selection

### 3.1 Data sources

This study uses data from the CFPS, a comprehensive survey conducted by Peking University every 2 years. CFPS aims to capture the dynamic changes in Chinese society, including economic shifts, demographic changes, and evolving social conditions at different levels: individual, familial, and community. For this research, the latest three rounds of tracking survey data from the CFPS in 2018, 2020, and 2022 were selected. By identifying respondents through their personal codes, a consistent sample of residents across these three periods was compiled into panel data. Using panel data helps mitigate potential issues of estimation accuracy due to small sample sizes (37). Following the selection process, each period yielded 2,120 valid data points, culminating in a comprehensive dataset comprising 6,360 observations.

### 3.2 Model specification

#### 3.2.1 Baseline regression model

In order to explore the impact of digital economy development on respondents’ SWB, based on the previous theoretical analysis, this article constructs the following benchmark regression model (Equation 1) (9, 10):


(1) $$\mathrm{Ha}p_{\mathrm{ijt}}=\alpha +\mathrm{\beta Di}g_{\mathrm{ijt}}+\gamma X_{\mathrm{ijt}}+u_{j}+v_{t}+\varepsilon_{\mathrm{ijt}}$$

The explanatory variable is $\mathrm{Ha}p_{\mathrm{ijt}}$ , which denotes the SWB score of individual i in province j at time *t*; the core explanatory variable *Dig* denotes the comprehensive level of individual digital economy utilization; and *X* is a series of control variables including respondents’ individual characteristic information variables. $u_{t}$ and $v_{t}$ represent regional and time fixed effects respectively, and $\varepsilon_{\mathrm{ijt}}$ represents the error term.

#### 3.2.2 Mediation effect models

To effectively identify the pathways through which the digital economy influences SWB, this study follows the approach of Shi et al. (11) to constructs a transmission effects model as shown in Equations 2 and 3:

Step 1: Test the effect of digital economy on the mediating variables (income, health, life quality, environmental quality, and governance effectiveness):


(2) $$M_{\mathrm{ijt}}=\alpha +\mathrm{\beta Di}g_{\mathrm{ijt}}+\gamma X_{\mathrm{ijt}}+u_{j}+v_{t}+\varepsilon_{\mathrm{ijt}}$$

Step 2: Test the effect of mediating variables on SWB:


(3) $$\mathrm{Ha}p_{\mathrm{ijt}}=\alpha +\mathrm{\beta Di}g_{\mathrm{ijt}}+\theta M_{\mathrm{ijt}}+\gamma X_{\mathrm{ijt}}+u_{j}+v_{t}+\varepsilon_{\mathrm{ijt}}$$

### 3.3 Variable selection and definition

#### 3.3.1 Dependent variable

We primarily employ the question “How happy are you?” from the questionnaire. Responses ranging from 1 to 10 are used to assess the respondents’ SWB (17). For robust regression analysis, we process the happiness scores as follows:

- We categorize respondents as “unhappy” if their scores are below 4 and “happy” if their scores are 4 or above.
- Alternatively, we combine neighboring scores and divide them into five levels: “very unhappy,” “unhappy,” “average,” “happy,” and “very happy.”
- We also directly use the response to the question “How happy are you?”

#### 3.3.2 Core independent variable

Drawing on the multifaceted nature of the digital economy, we begin with the areas of digital technology application that people incorporate into their daily lives in the context of the digital economy. In alignment with the CFPS survey questionnaire, questions about digital technology usage are structured around “digital device utilization, digital applications, digital dependency, and digital information access.” By also considering the digital atmosphere of the region, a comprehensive indicator system for personal digital economy utilization is developed, consisting of 5 primary indicators and 24 secondary ones (12). The entropy method was used to objectively assign weights to the fundamental indicators of the digital economy, culminating in the calculation of the respondents’ level of personal digital economy utilization via the linear weighting approach. For a detailed overview, see Table 1.

**Table 1:** Individual-level utilization of the digital economy.

Primary index	Secondary index	Index description
Digital device utilization	Whether or not you use a cell phone	Yes = 1, No = 0
	Whether or not you use a cell phone to access the Internet	Yes = 1, No = 0
	Whether or not you use a computer to access the Internet	Yes = 1, No = 0
Digital applications	Frequency of use of the Internet for learning	Number of uses per week
	Frequency of using the Internet for work	Number of uses per week
	Frequency of use of the Internet for socializing	Number of uses per week
	Frequency of use of the Internet for entertainment	Number of uses per week
	Frequency of Internet business activity	Number of uses per week
	Receive and send e-mail	Number of incoming and outgoing mailings per week
Digital dependency	Importance of work while online	Importance rating 1–5
	Importance of socializing while online	Importance rating 1–5
	Importance of entertainment when surfing the Internet	Importance rating 1–5
	Importance of business activities while online	Importance rating 1–5
Digital information access	The importance of television as a channel of information	Importance rating 1–5
	The extent to which the Internet is important as a channel for information	Importance rating 1–5
	The importance of broadcasting as a channel of information	Importance rating 1–5
	The extent to which cell phone text messaging is important as an information channel	Importance rating 1–5
Digital living environment	Internet penetration	Number of Internet users as a percentage of resident population
	Telephone penetration rate	Ratio of landlines and cell phones to total population in administrative areas
	Size of the radio and television industry	Number of listed companies in radio, television, film and audiovisual production
	Digital industry practitioners	Average number of employees at the end of the year in the information transmission, software and information technology services industry
	Digital Inclusive Finance Index	Peking University Digital Inclusive Finance Index
	Level of digital e-government	Number of government websites
	Number of new government media	New Media Platform Government Official Account

Due to space limitations, detailed results are not presented. All indicators are positive factors.

#### 3.3.3 Control variables

Relevant studies have demonstrated the importance of individual characteristic variables in influencing residents’ SWB (15, 17). Therefore, this article focuses on incorporating control variables like gender, age, years of education, marital status, employment status, and health status of respondents in the CFPS program into the regression equation. For a comprehensive analysis, Table 2 presents descriptive statistics for each variable.

**Table 2:** Descriptive statistics of main variables.

Variable	Characterization method	Mean	SD
Happiness 1	0 = unhappy, 1 = happy	0.8738	0.1867
Happiness 2	1–5 are very unhappy, unhappy, average, happy, and very happy, respectively	3.9444	0.9199
Happiness 3	On a scale of 1–10, respondents’ answers reflect their actual happiness level	7.4703	2.0443
Health situation	unhealthy = 1, average = 2, relatively healthy = 3, healthy = 4, very healthy = 5	3.2248	1.0833
Digital economy	Digital device use, digital applications, digital dependencies, digital information access, digital living environment	11.8874	4.1402
Age	Respondents’ actual age in the survey year	38.5245	11.359
Gender	Male = 0, Female = 1	0.4021	0.4903
Marital status	Unmarried = 0, Married = 1	0.7984	0.4012
Education	Respondents’ actual years of education	9.2958	4.0502
Work	Not employed = 0, employed = 1	0.7281	0.4449
Income	Gross personal income (Wan Yuan)	3.2728	3.8577
Insurance	No = 0, Yes = 1	0.6925	0.4616
Body mass index	Weight/height^2	21.8096	3.2079
Physical exercise	Weekly exercise frequency	3.5093	0.7376
Registration information	Agricultural households = 0, non-agricultural households = 1	0.4139	0.4641

Table 2 indicates that 12.62% of the residents surveyed in the CFPS expressed unhappiness, while 87.38% were happy. Among those who were happy, 40.37% said they were “happy,” and 30.64% said they were “very happy.” Overall, the respondents’ SWB is high, with an average score of 7.47, which is considered medium-high. This indicates that most respondents are satisfied with their lives.

## 4 Empirical results and analysis

### 4.1 Benchmark regression results

To ensure the robustness of the regression results, this study applies different methods of regression analysis according to how the dependent variable, the respondent’s happiness, is characterized. For a binary categorization of “unhappiness” and “happiness,” we employ the logit method for empirical analysis. When happiness is categorized into the following five levels: “very unhappy,” “unhappy,” “average,” “happy,” and “very happy,” we employ the ordered probit method. When using actual scores from 1 to 10 to measure SWB, we conduct empirical analysis using the panel data fixed effects method after passing the Hausman test (*p* < 0.01). The results examining the impact of digital economy development on residents’ SWB are presented in Table 3.

**Table 3:** Benchmark regression results.

Variable	Logit method		Ordered probit method		Fixed effects method	
	(1)	(2)	(3)	(4)	(5)	(6)
Digital economy	0.6985^***^	0.6985^***^	0.1319^***^	0.1078^***^	0.2654^***^	0.2176^***^
	(10.40)	(7.29)	(10.15)	(5.72)	(10.90)	(6.41)
Health situation		0.3919^***^		0.2158^***^		0.3865^***^
		(7.87)		(12.78)		(12.90)
Educational level		0.0568^***^		0.0064^*^		0.0163^**^
		(3.08)		(1.66)		(2.35)
Age		−0.1162^***^		−0.0745^***^		−0.1258^***^
		(−3.18)		(−10.35)		(−9.82)
Age squared		0.0016^***^		0.0009^***^		0.0015^***^
		(3.78)		(10.44)		(10.11)
Gender		0.2529		0.0686		0.1346
		(1.60)		(1.18)		(1.16)
Registration information		0.1028		0.0230		0.0091
		(0.69)		(0.85)		(0.19)
Marital status		1.0610^***^		0.4256^***^		0.8147^***^
		(8.12)		(14.31)		(15.11)
Work situation		−0.0397		0.0179		0.0333
		(−0.32)		(0.67)		(0.69)
Income level		0.0567		0.0935^***^		0.1538^***^
		(0.39)		(3.03)		(2.76)
Insurance situation		0.0884		0.0359		0.0645
		(0.76)		(1.46)		(1.45)
Body mass index		−0.0199		−0.0174^***^		−0.0340^***^
		(−0.97)		(−3.67)		(−3.96)
Physical exercise		0.2054^*^		0.0993^***^		0.1965^***^
		(1.75)		(4.39)		(4.81)
Constant term	2.2037^***^	−0.2762			7.0011^***^	6.5634^***^
	(21.19)	(−0.29)			(14.28)	(17.42)
Regional fixed					Yes	Yes
Time fixed					Yes	Yes
*N*	6,360	6,360	6,360	6,360	6,360	6,360

*t*-values in parentheses; *p < 0.1, **p < 0.05, ***p < 0.01.

Table 3 displays the results of our regression analyses. Columns (1) and (2) illustrate the outcomes using the logit method, where column (1) details results based on core explanatory variables alone, and column (2) includes results after accounting for additional individual characteristic variables. Columns (3) through (6) present results from applying the ordered probit method and the panel data fixed effects method, respectively. These findings collectively suggest that the digital economy significantly boosts residents’ SWB, a trend that is consistent across various econometric regression analyses and regardless of the inclusion of individual characteristic variables for control.

Specifically, referencing the panel data fixed effects models’ results in column (6), a one-unit increase in the level of digital economy development results in a 0.2176 unit increase in respondents’ SWB. Against China’s “Digital China” initiative and the deep integration of digital tools (e.g., mobile payments, telemedicine) into daily life, the digital economy boosts surveyed residents’ SWB by cutting transaction costs, expanding service access, and optimizing public efficiency—addressing practical needs and aligning with the national digital welfare goals (15–17). The results for control variables show that respondents’ SWB increases significantly with improved health and education, further emphasizing the importance of these factors in enhancing residents’ SWB. A U-shaped relationship between age and residents’ SWB was observed, indicating that well-being decreases as individuals transition from a carefree school life to responsibilities like work, marriage, children, and mortgage payments (11). However, as their children grow up and start families, the pressure on middle-aged and older adults respondents reduces, leading to an increase in their sense of well-being. SWB is not related to gender. Married respondents reported higher SWB compared to unmarried ones. Income level was significantly and positively correlated with individual SWB. A higher body mass index was linked to lower SWB, possibly due to the negative self-esteem associated with obesity. Engaging in sports activities significantly improved the SWB of the interviewed residents. Sports participation not only enhances physical health but also facilitates social interactions and satisfaction (22). Registered residence information (Hukou status), employment status, and insurance situation did not impact the SWB of the residents in this study.

### 4.2 Robustness tests

#### 4.2.1 Propensity score matching

In the baseline regression analysis, this article used multiple measurement methods to ensure the reliability of results. However, the impact of digital economic development on individuals’ SWB could be affected by personal characteristics and other factors, leading to potential biases. To address this, we employed propensity score matching (PSM) to address endogeneity issues related to observable variables. PSM has been used to construct a counterfactual analysis framework that effectively addresses endogeneity issues caused by observable variables (38). We selected key control variables like education level, age, gender, household registration status (Hukou), marital status, employment, insurance status, and income to estimate propensity scores. After a balance test based on PSM data requirements, we used nearest-neighbor matching with a 1:2 ratio based on propensity scores. The outcomes, including the results of the smoothness test, are presented in Table 4.

**Table 4:** Smoothness tests.

Variable	Matching status	Mean		% bias	% reduction bias	*t*-test	
		Treated	Control			*t*	*p*-value
Edu	U	11.87	8.0081	109.2		50.76	0.000
	M	11.87	11.886	−0.5	99.6	−0.20	0.841
Age	U	33.076	41.248	−79.1		−36.36	0.000
	M	33.076	33.088	−0.1	99.6	−0.05	0.960
Gender	U	0.4543	0.37598	15.9		7.62	0.000
	M	0.4543	0.44575	1.7	89.1	0.71	0.479
Registration (Hukou)	U	0.43131	0.25527	37.7		18.33	0.000
	M	0.43131	0.44222	−2.3	93.8	−0.91	0.365
Marital	U	0.70077	0.84716	−35.5		−17.61	0.000
	M	0.70077	0.70784	−1.7	95.2	−0.64	0.523
Work	U	0.875	0.65468	53.8		24.21	0.000
	M	0.875	0.8691	1.4	97.3	0.73	0.467
Insure	U	0.74351	0.66647	17		7.96	0.000
	M	0.74351	0.75206	−1.9	88.9	−0.81	0.418
Incomes	U	4.5989	4.388	54.6		25.63	0.000
	M	4.5989	4.5957	0.8	98.5	0.38	0.706

In the table, “U” denotes the unmatched sample and “M” the matched sample. “% bias” signifies the standardized bias, whereas “% reduction bias” indicates the percentage reduction in bias achieved through matching.

Table 4 presents the results of the smoothness test. According to the table, the standardized deviation of the control variables is significantly reduced after matching, with all deviations falling under 10%. This demonstrates that the matching process effectively balances the differences between the experimental and control groups, and the results passed the balance test. Table 5 displays the regression results of the average treatment effect.

**Table 5:** Results of average treatment effects.

Matching method	Matched method	ATT	Standard error	*t*-value
Nearest-neighbor	U	0.3475^***^	0.0429	8.11
	M	0.1073^***^	0.0385	2.79
Kernel matching	U	0.3475^***^	0.0429	8.11
	M	0.2005^***^	0.5457	3.67
Radius matching	U	0.3475^***^	0.0429	8.11
	M	0.3511^***^	0.0590	5.59

***p < 0.01. Results of kernel-matching and radius-matching methods are omitted due to space limitations. “U” denotes the unmatched sample and “M” the matched sample.

Based on the regression results in Table 5, the application of nearest-neighbor matching, kernel matching, and radius matching methods to control variables in tests indicates that the impact of digital economic development on enhancing the SWB of surveyed residents remains unchanged. This further validates the robustness of the previous findings.

#### 4.2.2 Additional robustness checks

To further verify the robustness of the regression results, this study employs the following methods for additional validation: (1) re-measuring the development level of the digital economy using the Principal Component Analysis (PCA) method; (2) re-conducting validation with two alternative metrics for SWB, namely life satisfaction (“How satisfied are you with your current life? 1–5 scale”) and affective balance—where affective balance is calculated as the difference between positive emotions (measured by “frequency of happiness in the past month”) and negative emotions (measured by “frequency of sadness”); (3) analyzing the lagged one-period data of the explanatory variable (digital economy development level) to explore the dynamic effects of digital economy development; (4) excluding samples from regions with a relatively developed digital economy in China (e.g., Beijing, Shanghai, and Zhejiang) and re-performing the regression analysis. The detailed regression results are presented in Table 6.

**Table 6:** Results of robustness test.

Variable	Re-measure digital economy	Alternative SWB metric	Affective balance	Ordinary cities	Lagged-digital economy
	(1)	(2)	(3)	(4)	(5)
Digital economy	0.3813^***^	0.7232^***^	0.6842^***^	0.1712^***^	0.0873^**^
	(4.09)	(5.87)	(4.14)	(3.98)	(2.49)
Control variables	Yes	Yes	Yes	Yes	Yes
Regional fixed	Yes	Yes	Yes	Yes	Yes
Time fixed	Yes	Yes	Yes	Yes	Yes
*N*	6,360	6,360	6,360	5,160	2,120

*t*-values in parentheses; **p < 0.05, ***p < 0.01.

All results in Table 6 indicate that the development of the digital economy can effectively enhance residents’ SWB, confirming the robustness of the study’s findings.

### 4.3 Addressing endogeneity

In regression analysis examining the digital economy’s impact on SWB, endogeneity issues may arise from two key sources: reverse causality and omitted variables. Reverse causality occurs because individuals with higher SWB may be more inclined to seek new experiences enabled by digital technologies (e.g., proactively trying online services or digital tools), thereby stimulating market demand for digital products and creating a feedback loop where SWB influences engagement with the digital economy rather than the other way around. Omitted variables—such as unobserved regional cultural attitudes toward technology adoption or unmeasured levels of individual adaptability to digital tools—could also simultaneously affect both an individual’s participation in the digital economy and their SWB. To mitigate these endogeneity issues, this study employs the two-stage least squares (2SLS) method and selects instrumental variables (IVs) that satisfy the core criteria of relevance and exogeneity—a common strategy in microdata research, where researchers use aggregated regional-level data to instrument (39).

Four IVs are introduced to capture the exogenous variation in regional digital economy development, with their relevance justified by the path dependence of digital infrastructure (9, 10, 12, 19): (1) The number of post offices per province at the end of 1984—As historical postal and telecommunications infrastructure, post offices laid the foundation for regional communication networks; areas with more post offices in 1984 had pre-existing advantages in building later digital communication systems, creating a positive correlation with current digital economy levels; (2) The number of fixed-line telephones per 100 residents per province at the end of 1984—Fixed-line telephone networks were the core of early information transmission; higher penetration in 1984 reflects stronger regional communication infrastructure, which directly facilitated the subsequent rollout of digital technologies; (3) The provincial Internet penetration rate in 2005—As a mid-term historical indicator, this predates the large-scale development of the modern digital economy and reflects early regional digitalization momentum; the indicator’s path-dependent growth ensures a stable correlation with current digital economy development; (4) The average regional ICT infrastructure level—Measured by household computer and mobile phone ownership rates in the respondent’s region, this variable captures peer effects and infrastructure constraints in digital adoption; individuals’ engagement with the digital economy depends on the availability of regional ICT tools, while the aggregate level of such infrastructure is exogenous to individual SWB.

A critical strength of the selected IVs lies in their exogeneity, particularly driven by historical path dependence, which ensures that they do not directly affect current SWB or correlate with unobserved confounding factors. First, the temporal exogeneity of historical data: The 1984 post office count, 1984 fixed-line telephone penetration, and 2005 Internet penetration are all historical indicators that predate the mature development of the modern digital economy. Since current individual SWB cannot retroactively influence infrastructure decisions made decades ago, reverse causality between the IVs and SWB is completely eliminated—this addresses concerns about historical path dependence by leveraging “time-asymmetry” to break endogenous links. Second, the absence of direct effects on SWB: The IVs only influence SWB through their impact on current digital economy development. For example, 1984 post office counts do not directly affect an individual’s happiness today; their sole role is to shape the regional digital infrastructure that enables individuals’ current digital economy engagement. Similarly, regional ICT infrastructure affects SWB not through inherent attributes, but by facilitating individuals’ access to digital services (e.g., telemedicine, online socialization) (9).

Relevance is confirmed by first-stage tests: The WALD *F*-statistic far exceeds the critical value of 10, rejecting weak IV concerns; the LM statistic confirms joint significance of IVs, validating their relevance to the endogenous digital economy variable. The 2SLS regression results using the above IVs are detailed in Table 7, confirming that the core finding—the digital economy’s positive impact on SWB—remains robust after addressing endogeneity.

**Table 7:** Results of the instrumental variable method test.

Instrumental variable	Post office	Fixed–line telephone	Internet penetration	ICT infrastructure
	(1)	(2)	(3)	(4)
Digital economy	0.8449^***^	0.5865^***^	0.3581^***^	0.8715^***^
	(3.17)	(4.54)	(2.79)	(3.36)
First-stage regression coefficient	0.0169^***^	0.0690^***^	0.0141^***^	0.4651^***^
	(4.80)	(2.97)	(4.31)	(8.51)
LM	0.0010	0.0000	0.0002	0.0040
Wald *F*	33.22	34.954	41.588	51.371
Control variable	Yes	Yes	Yes	Yes
Regional fixed	Yes	Yes	Yes	Yes
Time fixed	Yes	Yes	Yes	Yes
*N*	6,360	6,360	6,360	6,360

All entries in this table use SWB as the dependent variable; the row labels correspond to the instrumental variables employed. *t*-values in parentheses; ***p < 0.01. The Kleibergen-Paap rk LM statistic (for each instrumental variable) is the *p*-value for the under-identification test, and the Cragg-Donald Wald *F* statistic is the 10% critical value for the StockYogo weak identification test.

### 4.4 Heterogeneity analysis

#### 4.4.1 Heterogeneity analysis by individual characteristics

In this section, based on the mechanism analysis presented earlier, we focus on exploring the heterogeneous effects of the digital economy on residents’ SWB across different factors, including age, digital literacy, and frequency of digital tool use. We classify samples according to the following method: (1) Age Group (minors <30, middle-aged 30–59, older adults >60); (2) Digital Literacy (proxied by education level: college+ = high literacy, middle school− = low literacy); and (3) Digital Usage Frequency (low < average level, high ≥ average level).

Table 8 presents regression results on how digital economic development influences individuals’ SWB, shedding light on heterogeneities across age, education, digital usage frequency, and other characteristics.

**Table 8:** Regression results of heterogeneity analysis by individual characteristics.

Variable	Age			Digital literacy		Usage frequency	
	Minors	Middle-aged	Older Adults	Low	High	Low	High
	(1)	(2)	(3)	(4)	(5)	(6)	(7)
Digital economy	0.0832	0.2183^***^	0.0952^*^	0.1423^**^	0.2313^***^	0.0512	0.2902^***^
	(1.41)	(5.87)	(1.9)	(2.44)	(7.01)	(0.83)	(4.28)
Control variables	Yes	Yes	Yes	Yes	Yes	Yes	Yes
Regional fixed	Yes	Yes	Yes	Yes	Yes	Yes	Yes
Time fixed	Yes	Yes	Yes	Yes	Yes	Yes	Yes
*N*	1,120	3,420	1820	3,600	2,760	1,040	5,320

*t*-values in parentheses; *p < 0.1, **p < 0.05, ***p < 0.01. Results for remaining control variables are omitted due to space constraints.

Results for Minors (column 1), Middle—aged (column 2), and Older Adults (column 3) reveal age—driven differences. The digital economy exerts a significant positive impact on the SWB of middle—aged adults but not for minors or the older adults. Minors, with their unformed life outlooks and limited social engagement, have a narrow understanding of how the digital economy relates to happiness. Older adults individuals, facing declines in physical function, prioritize health and family support, reducing the digital economy’s influence on their SWB (28). In contrast, middle—aged adults, deeply embedded in social and economic activities, better leverage the benefits of the digital economy, confirming H6a—the positive effect is stronger for middle—aged adults (30–59 years) than for minors (<30 years) and the older adults (>60 years).

Columns 4 (Low digital literacy), and 5 (High digital literacy) show consistency in the digital economy’s positive impact across educational levels. While the hypothesis predicted a stronger effect for high—educated groups, the results instead highlight the digital economy’s universal benefit. Its attributes of universality, convenience, and popularity transcend educational boundaries—individuals with varying education levels (elementary, high school, college or above) all gain well—being from it, such as accessing information, boosting work efficiency, or seeking business opportunities (29). Though not directly supporting “stronger for high—educated,” it underscores the digital economy’s inclusive nature, with the positive effect present across education groups, providing partial insights into H6b.

Columns 6 (Low frequency) and 7 (High frequency), grouped by the mean of digital usage frequency, show distinct effects: the digital economy has no significant impact on SWB for low—frequency users (who are not deeply engaged in the digital ecosystem, miss out on benefits like efficient information access or income channels), yet exerts a significant positive effect on high—frequency users. The latter, via extensive engagement, access diverse resources, partake in digital—enabled activities (e.g., online entrepreneurship), and enjoy smart services, enhancing their SWB (26). This confirms H6c—the positive effect is stronger for high—frequency users, versus no significant impact for low-frequency users.

#### 4.4.2 Heterogeneity analysis by compositional dimensions of the digital economy

The previous analysis clearly shows that the development of the digital economy significantly impacts the SWB of the surveyed residents (22). In this article, we primarily use secondary indicators like digital device utilization, digital applications, digital dependency, digital information access, and the digital living environment as variables to explore the diverse effects of digital economy development on the SWB of the surveyed residents in each subdimension of the digital economy.

The results in Table 9 reveal heterogeneous effects of different digital economy dimensions on residents’ SWB, with clear distinctions between positive drivers and negative factors. Specifically, the regression coefficient for digital device utilization (e.g., ownership of mobile phones or computers) is statistically insignificant, indicating that mere access to digital devices does not directly improve residents’ SWB. In contrast, three dimensions exert significant positive impacts at the 1% significance level: digital applications (e.g., weekly internet use for work, learning, or daily services), digital information access (e.g., relying on the internet/TV for key information), and the digital living environment (e.g., regional internet penetration rates, digital public service coverage). These findings confirm that in the digital economy era, SWB improvement depends not on “having digital tools” but on “effectively using digital resources”: digital information access and the digital living environment enhance convenience in daily life and work by reducing transaction costs and overcoming time and space barriers, while their social attributes (e.g., online communities, interactive platforms) promote interpersonal interaction, alleviate social isolation among residents, and strengthen communication—ultimately fostering closer social bonds and boosting overall well-being; digital applications further enrich life experiences, such as expanding entertainment options and enabling accessible online education, which directly increase pleasure and contribute to SWB (9, 25, 26, 28).

**Table 9:** Regression results of heterogeneity analysis by compositional dimensions of the digital economy.

Variable	Digital device utilization	Digital applications	Digital dependency	Digital information access	Digital living environment
	(1)	(2)	(3)	(4)	(5)
Sub-dimensional	−0.0163	0.0436^***^	−0.0665^***^	0.3347^***^	0.7843^***^
	(−0.12)	(4.39)	(−2.86)	(12.23)	(4.45)
Control variables	Yes	Yes	Yes	Yes	Yes
Regional fixed	Yes	Yes	Yes	Yes	Yes
Time fixed	Yes	Yes	Yes	Yes	Yes
*N*	6,360	6,360	6,360	6,360	6,360

*t*-values in parentheses; ***p < 0.01. Results for remaining control variables are omitted due to space constraints.

Notably, digital dependence (e.g., excessive reliance on digital tools for individuals’ daily activities) exerts a significant negative effect on SWB. This aligns with evidence that excessive digital engagement suppresses individual happiness through multiple mechanisms: it induces anxiety and depression via information overload or upward social comparisons, weakens the quality of real-life social interactions by replacing in-person communication with superficial online exchanges, exacerbates work-life imbalance (blurring boundaries between online work and offline rest), and compromises physical and mental health (e.g., sleep deprivation from late-night device use, chronic sedentary behavior-related diseases) (12). These negative impacts are particularly pronounced among individuals who rely heavily on digital tools for social interaction, information processing, and daily task management, as their lives become overly intertwined with digital environments, leaving them little room for offline recovery and emotional regulation (21).

### 4.5 Analysis of transmission pathways

Based on the mechanism analysis from the previous section, this article examines the role of income level, health status, life quality, living environment, and government effectiveness as mediating variables in the influence of digital economy development on SWB among respondents. For income level, instead of using personal income from the original questionnaire (which was designated as a control variable in previous regression analyses), we use the survey question “severity of the wealth gap in China” to represent the overall economic conditions. The health pathway is operationalized using an overall health measure that incorporates mental health survey questions such as satisfaction with life and overall happiness. Life quality is assessed by the question, “How satisfied are you with your life?” The living environment is characterized by the quality of the environment in the respondents’ residential areas. Government effectiveness in these areas is assessed by combining scores from questions about various issues (e.g., employment, education, healthcare, housing, social security, and government corruption) in China. Any negative scores in these questions are normalized to positive values.

This article employs the mediation effect models (2) and (3) introduced earlier to examine the transmission pathway, and adopts the Bootstrap method for mediation effect testing. The Bootstrap method enhances statistical validity by recalculating precise standard errors through resampling with replacement over a specified number of iterations (40). The Bootstrap sample size is set at 2000, with a 95% confidence interval constructed. The regression results pertaining to the mediation effect are presented in Table 10.

**Table 10:** Regression results of transmission pathways.

Variable	Direct effect	Indirect effect	95% CI (lower)	95% CI (upper)	Indirect/total effect
Income level	0.1892^***^	0.0148^***^	0.15136	0.28316	0.0726
	(5.41)	(3.49)			
Health status	0.1563^***^	0.2019^***^	0.01692	0.02643	0.5637
	(2.76)	(3.65)			
Life quality	0.1922^***^	0.1062^***^	0.00141	0.01768	0.3559
	(8.90)	(5.25)			
Environmental quality	0.2013^***^	0.0040^***^	0.04460	0.08081	0.0195
	(4.33)	(3.92)			
Government effectiveness	0.1684^***^	0.0814^***^	0.21261	0.38590	0.3259
	(4.73)	(3.73)			

This table was constructed by the authors. “95% CI (lower)” and “95% CI (upper)” denote the lower and upper bounds of the 95% confidence interval obtained from 2,000 bootstrap replications, while “Indirect/total effect (%)” reports the share of the total effect attributable to the indirect pathway. *t*-values in parentheses; ***p < 0.01. Results for remaining control variables are omitted due to space constraints.

The validity of a mediation effect is confirmed when the 95% confidence interval for the indirect effect excludes zero (40), and the regression results in Table 10 show that in the pathway connecting the digital economy to respondents’ SWB, five factors—namely income level, health status, life quality, environmental quality, and government effectiveness—play significant indirect roles, with the direct effects, indirect effects, and the ratio of indirect effects to total effects explicitly reported for each mediator. Specifically, for the income level pathway, the direct effect of the digital economy on SWB is 0.1892 (*t* = 5.41), the indirect effect (via income level) is 0.0148 (*t* = 3.49), and the indirect effect accounts for 7.3% of the total effect [i.e., calculated as the indirect effect (0.0148) divided by the total effect (0.1892 + 0.0148 = 0.2040)]; for the health status pathway, the direct effect is 0.1563 (*t* = 2.76), the indirect effect is 0.2019 (*t* = 3.65), and the indirect effect accounts for 56.4% of the total effect; for the life quality pathway, the direct effect is 0.1922 (*t* = 8.90), the indirect effect is 0.1062 (*t* = 5.25), and the indirect effect accounts for 35.6% of the total effect; for the environmental quality pathway (characterized by perceived residential environmental quality), the direct effect is 0.2013 (*t* = 4.33), the indirect effect is 0.0040 (*t* = 3.92), and the indirect effect accounts for 2.0% of the total effect; for the government effectiveness pathway, the direct effect is 0.1684 (*t* = 4.73), the indirect effect is 0.0814 (t = 3.73), and the indirect effect accounts for 32.6% of the total effect. Among these mediators, health status is the most impactful (56.4% of the total effect), followed by life quality (35.6%) and government effectiveness (32.6%), which suggests that the digital economy enhances SWB primarily by improving individuals’ physical and mental health—consistent with Yang and Hu’s (23) finding that digital health literacy boosts well-being. These findings collectively confirm that digital economic development enhances SWB by reducing economic disparities, improving health and life quality, enhancing environmental quality, and promoting government effectiveness, thereby supporting the Hypotheses 1–5 proposed in the study.

## 5 Discussion and policy implications

### 5.1 Conclusion

Against the backdrop of China’s deepening digital transformation, this study draws on three waves of panel data from the China Family Panel Studies (CFPS, 2018, 2020, 2022) and anchors its analysis in the Capability Approach and the Digital Divide Theory. By employing a series of econometric methods (including panel models, instrumental variable estimation, and robustness tests), it systematically examines the impact of the digital economy on residents’ SWB, alongside its underlying mechanisms and heterogeneous effects. The core findings are as follows: First, the digital economy exerts a stable and significant positive effect on residents’ SWB—this conclusion remains robust even after addressing endogeneity issues such as reverse causality and omitted variables and conducting multiple robustness checks (e.g., propensity score matching, alternative variable measurements). Second, mechanism analysis reveals five mediating pathways with distinct importance: health status is the most critical mediator (accounting for 56.4% of the total effect), followed by life quality (35.6%) and government effectiveness (32.6%), while income level (7.3%) and environmental quality (2.0%) play relatively minor roles. This indicates the digital economy enhances SWB primarily by improving physical and mental health (e.g., via digital health literacy and telemedicine), enriching individuals’ daily life experiences (e.g., convenient online services, accessible knowledge), and optimizing public service access (e.g., efficient digital government platforms). Third, dimensional and group-level heterogeneities are evident: digital applications, digital information access, and the digital living environment significantly boost SWB, whereas digital dependence exerts a negative effect by inducing anxiety, weakening offline social interaction, and harming physical health; meanwhile, vulnerable groups such as the older adults and minors face severe digital divide-related barriers, which exacerbate inequalities in SWB.

### 5.2 Research limitations

While this study provides insights into the digital economy-SWB relationship, it has three notable limitations that warrant attention. First, the subjectivity in measuring mediating variables may compromise the precision of mechanism analysis. Key variables such as “income level” (proxied by respondents’ perceived severity of the wealth gap) and “health status” (based on self-assessed health scores) rely on subjective self-reports, which could be biased by individual cognitive differences, such as overly optimistic residents overestimating their health status. Additionally, the unavailability of objective indicators in the CFPS—such as regional per capita GDP (to replace perceived wealth gaps), official environmental pollution indices (to replace the self-reported environmental quality), or medical records (to replace self-assessed health)—further limits the robustness of the mediation results. Second, sample representativeness has room for improvement. Although CFPS data is nationally representative, the analysis focuses solely on registered urban and rural residents in China, with no explicit discussion of special groups such as rural–urban migrants or regional policy heterogeneities (e.g., divergent digital economy support policies in the eastern vs. western regions of China). Migrants often face unique digital access barriers (e.g., limited access to local digital public services), and regional policy differences may amplify or weaken the digital economy’s impact on SWB, reducing the external generalizability of the study’s conclusions. Third, the analysis of negative effects and vulnerable groups is insufficient. While the study identifies digital dependence and the digital divide as negative factors, it lacks in-depth exploration of the specific mechanisms (e.g., how digital dependence affects sleep quality or mental health) and fails to systematically examine the challenges faced by vulnerable groups such as the older adults (e.g., digital literacy deficits) and low-skilled workers (e.g., limited access to digital employment opportunities). This limits the study’s ability to provide targeted policy guidance for addressing these issues. For future research, integrating objective data (e.g., official economic and environmental indicators, medical records) and expanding the sample to include migrant populations will help enhance the external generalizability of conclusions.

### 5.3 Policy implications

To maximize the SWB-promoting effects of the digital economy and address the aforementioned limitations, three sets of targeted policy recommendations are proposed. First, optimize key mediating pathways to amplify positive impacts, with a focus on health and life quality. Specifically, expand telemedicine coverage in rural and remote areas, develop user-friendly digital health tools such as the simplified health management apps, and popularize digital health literacy through community-based training programs (especially for middle-aged and older adults groups) to strengthen the health-mediated effect of the digital economy. Simultaneously, support inclusive digital services such as low-cost online education platforms and affordable digital devices and improve the digital living environment (e.g., full 5G coverage in residential areas, smart community infrastructure) to further enrich residents’ daily life experiences. Second, narrow the digital divide to protect vulnerable groups. Promote the “age-appropriate transformation” of digital products (e.g., simplified mobile payment interfaces, voice-activated functions for the older adults) and provide offline support (e.g., community “digital assistants” to help the older adults with their digital operations) to increase the digital participation rate of the older adults. Launch free digital literacy training programs in migrant communities and rural areas, and integrate digital skills into vocational training programs for low-skilled workers to help them access digital employment opportunities. Additionally, allocate more digital infrastructure resources to the western and rural areas of China and unify digital public service standards for migrants (e.g., cross-regional recognition of digital health records) to reduce regional and group-based inequalities in digital access. Third, mitigate the negative effects of digital dependence. Launch public awareness campaigns on healthy digital use (e.g., advocating for limited screen time, balanced online-offline life) and encourage digital platforms to adopt “well-being-friendly” features (e.g., screen time reminders, night mode for eye protection) to guide residents toward their rational digital engagement.

## Data Availability

Publicly available datasets were analyzed in this study. This data can be found at: https://www.isss.pku.edu.cn/cfps/.
